# Research and Implementation of an Improved Non-Contact Online Voltage Monitoring Method

**DOI:** 10.3390/s26030782

**Published:** 2026-01-23

**Authors:** Meiying Liao, Jianping Xu, Wei Ni, Zijian Liu

**Affiliations:** 1 School of Electrical Engineering, Southwest Jiaotong University, Chengdu 611756, China; 2 College of Intelligent Manufacturing, Mianyang Teachers’ College, Mianyang 621000, China; 3 Key Laboratory of Magnetic Suspension Technology and Maglev Vehicle, Ministry of Education, Chengdu 611756, China; 4 Mianyang Weibo Electronics Co., Ltd., Mianyang 621000, China

**Keywords:** non-contact voltage measurement, RC input circuit, phase dithering method, stray capacitance, online monitoring

## Abstract

High-precision non-contact online voltage monitoring has attracted considerable attention due to its improved safety. Based upon existing research works and validation of non-contact voltage measurement techniques, an enhanced approach for online voltage monitoring is proposed in this paper. By analyzing the influence of the relationship between coupling capacitance and input capacitance on monitoring results, an RC-type signal input circuit with enhanced adaptability has been designed for practical engineering scenarios that may involve large input capacitance. Furthermore, a mixed-signal measurement method based on phase dithering is proposed to eliminate detection errors caused by relative phase drift during synchronous sampling in existing signal injection approaches. This improvement enhances measurement accuracy and offers a more robust theoretical basis for selecting injection signal frequencies. The hardware circuit architecture and data processing scheme presented in this work are straightforward and have been validated using an experimental prototype tested at 50 Hz/500 V and 2000 Hz/300 V. Long-term energized testing demonstrates that the system operates stably at room temperature with a relative measurement error below 0.5%. This study provides a high-precision, easily implementable non-contact measurement solution for online monitoring of low-frequency, low-voltage signals in complex electromagnetic environments such as industrial control signals, low-voltage power signals, and rail transit signals.

## 1. Introduction

Online monitoring of low-voltage signals is widely applied in power systems, rail transportation, and industrial control. Recent years have witnessed significant research advances in non-contact voltage measurement methods. Studies such as [1,2,3] have proposed non-contact voltage measurement approaches based on electric field coupling, enabling safer online voltage monitoring. Further application-oriented research using non-contact voltage measurement technology has been conducted in specific domains, including transformer voltage measurement, overhead line voltage measurement, and partial discharge detection, as documented in [4,5,6,7,8].

Conventional electric-field-coupling-based non-contact voltage measurement techniques are subject to considerable errors because the coupling capacitance is influenced by factors such as the insulation medium of the measured conductor, the sensor’s position, and the conductor diameter. In practical engineering applications, from manufacturing and transportation to installation and operation, dynamic variations in coupling capacitance inevitably arise due to differences in diameter or material between the measured and reference conductors, changes in the conductor’s position within the sensor aperture, and environmental temperature fluctuations. These variations introduce substantial measurement errors.

To address this issue, Yang et al. [9] proposed directly connecting the conductor core to the coupling probe to increase the conductive capacitance, thereby overwhelming the influence of coupling capacitance variations. Although effective, this method violates the principle of non-contact measurement and is unacceptable in safety-critical applications.References [10,11] proposed methods involving a paralleled large capacitor and establishing equation sets through topological switching of a known-impedance branch to solve for the unknown coupling capacitance, respectively. While paralleling a large capacitor can partially suppress the impact of dynamic coupling capacitance changes, it does not fundamentally eliminate the error, as such changes effectively alter the input signal; insensitivity to this variation implies insensitivity to the actual signal. The dynamic topology-switching approach, however, introduces additional capacitance from the switching devices into the signal path. Furthermore, it may lead to greater measurement errors if the coupling capacitance changes abruptly, such as due to vibration.

An effective approach to addressing this issue is the signal injection method proposed in studies such as [12,13,14,15,16]. The core principle of this method is to inject a reference signal of known frequency into the measurement loop, and then eliminate the influence of the coupling capacitance on the measurement result by solving the responses of both the reference signal and the measured signal. Shenil et al. conducted early feasibility research on a non-contact AC voltage measurement system based on signal injection, achieving high-precision voltage measurement through data analysis and processing on a host computer [12]. The work in [17] provides an in-depth analysis of the error and noise mechanisms of this method, laying a theoretical foundation for high-precision non-contact measurement. Building on this, Delle Femine et al. designed an integrated low-power sensor incorporating analog filtering and peak-detection circuits, which achieved high measurement accuracy [16] however, the circuit is relatively complex.

## 2. Research Status

**Figure 1:**
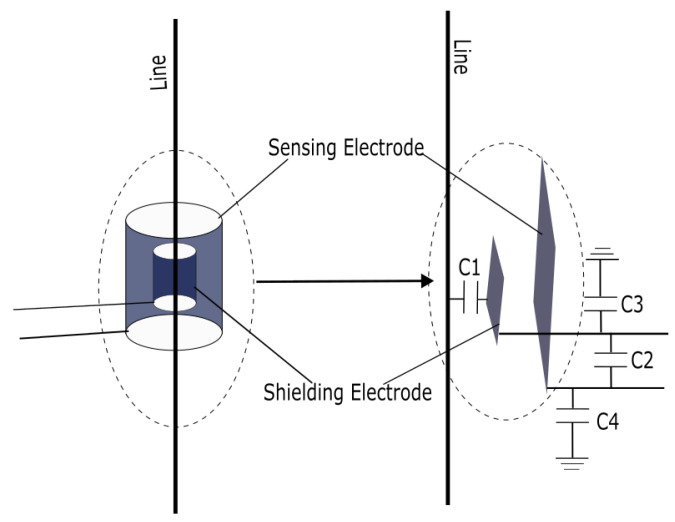
Equivalent Circuit Diagram of Capacitive Coupled Non-contact Voltage Measurement Probe.

In electric field coupling based voltage measurement, the coupling probe only needs to serve as an electrode capable of collecting electric charge and can take any geometric form. However, simulation results reported in [18] demonstrate that a cylindrical double-layer copper tube structure provides a more stable coupling capacitance compared with other electrode configurations. A schematic diagram of the probe and its equivalent capacitance distribution is presented in Figure 1.

### 2.1. Principle of the Signal Injection Method

**Figure 2:**
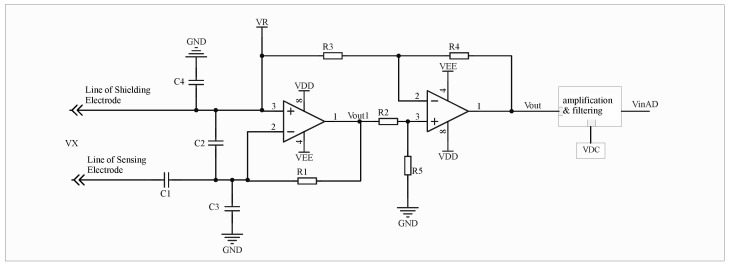
Equivalent Circuit of Signal Injection-Based Capacitive Coupled Non-contact Voltage Measurement.

By injecting a high-frequency signal, the coupling capacitance can be monitored in real time. The voltage on the insulated conductor is then determined by comparing the system’s responses to signals at different frequencies, thereby eliminating the influence of coupling capacitance variations. The principle of the equivalent circuit is illustrated in Figure 2.

In the circuit diagram, the first stage functions as an I–V conversion circuit, while the second stage subtracts $V_{R}$ from $V_{OUT1}$ to ensure that the subsequent conditioning circuits process only the effective signal associated with the coupling capacitance, thereby preserving a larger dynamic range for signal amplification.

In Figure 2, $C_{1}$ represents the coupling capacitance between the measured conductor and the sensing layer. Its value is jointly determined by factors such as the insulation medium of the measured line, the feed through position, and the electrode area. The measured signal is transmitted to the operational amplifier through $C_{1}$.

$C_{2}$ denotes the capacitance between the sensing layer and the shielding layer, which theoretically does not affect the measurement results.

$C_{3}$ corresponds to the input capacitance, including the equivalent distributed capacitance of the sensing layer and its connecting cable loop to the ground, as well as all stray capacitance s to ground along the transmission path. For the measured signal, $C_{3}$ acts as a load and therefore does not influence the measurement outcome. However, when the in-phase terminal of the operational amplifier is injected with the signal $V_{R}$, $C_{3}$ directly affects the measurement result of the injected signal.

$C_{4}$ represents the equivalent distributed capacitance of the shielding layer and its connected cable to the ground, which theoretically has no effect on the measurement results.

Let $V_{X}$ denote the measured voltage and $V_{R}$ the injected high-frequency signal voltage, with different amplitudes and angular frequencies. According to the circuit superposition theorem,

(1) $$V_{OUT}=K\omega_{x}C_{1}V_{x}R_{1}+K\omega_{R}\left(C_{1}+C_{3}\right)V_{R}R_{1}$$

where *K* denotes the amplification factor of the differential circuit. The output voltage $V_{OUT}$ contains two components with different frequencies, whose RMS values are $V_{OX}$ and $V_{OR}$, respectively. These RMS values can be obtained through appropriate filtering and computation of ${\dot{U}}_{OUT}$. According to Equation (1), we obtain:

(2) $$V_{OX}=K\omega_{x}V_{x}C_{1}R_{1}$$

(3) $$V_{OR}=K\omega_{R}V_{R}C_{1}R_{1}+K\omega_{R}V_{R}C_{3}R_{1}=V_{OR1}+V_{zero}$$

Let

(4) $$V_{OR1}=K\omega_{R}V_{R}C_{1}R_{1}$$

(5) $$V_{zero}=K\omega_{R}V_{R}C_{3}R_{1}$$

From Equation (5), it can be seen that if $C_{3}$ is extremely small, then $V_{zero}\approx 0$, at this point

(6) $$V_{OR}=V_{OR1}=K\omega_{R}V_{R}C_{1}R_{1}$$

If the value of $C_{3}$ is fixed, then under a given injection signal condition, $V_{zero}$ becomes a constant. This constant can be determined by setting the coupling capacitance $C_{1}=0$, i.e., by injecting only $V_{R}$ while positioning the sensing probe away from the measured conductor, and subsequently stored as a fixed zero reference in the register [13]. Now

(7) $$V_{OR2}=V_{OR}-V_{zero}=K\omega_{R}V_{R}C_{1}R_{1}$$

When the injection signal frequency and amplitude remain constant, both Equations (6) and (7) yield values that depend solely on $C_{1}$. By simultaneously solving Equations (2) and (6) (or Equation (7)), the measured signal voltage $V_{X}$ can be determined.

(8) $$V_{x}=\frac{V_{OX}\omega_{R}V_{R}}{V_{OR1}\omega_{x}}$$

or

(9) $$V_{x}=\frac{V_{OX}\omega_{R}V_{R}}{V_{OR2}\omega_{x}}$$

As shown in Equations (8) and (9), when this method is applied to measure $V_{X}$, its value remains independent of $C_{1}$, thereby effectively eliminating measurement errors caused by variations or uncertainties in the coupling capacitance.

### 2.2. Existing Issues

The signal injection method effectively mitigates the impact of coupling capacitance variations; however, it still faces two major challenges in engineering applications:

(1) Large distributed capacitance interference. Analysis of Equations (8) and (9) indicates that although the calculation formula for $V_{X}$ is not directly dependent on $C_{1}$, Equations (2) and (4) reveal that both $V_{OX}$ and $V_{OR}$ are influenced by $C_{1}$. In addition, $V_{OR}$ is also affected by the input capacitance $C_{3}$. Previous studies generally assume that $C_{3}$ is sufficiently small to be neglected when high measurement accuracy is not required, and this assumption has yielded satisfactory results under laboratory conditions. This conclusion is valid when considering only the discrete ground capacitance of the sensing probe and its connecting wires. However, when the non-contact voltage measurement method is applied to the low-voltage online monitoring scenarios discussed in this paper, the actual value of $C_{3}$ can become considerably larger due to the complex electromagnetic environment at engineering sites. To improve electromagnetic compatibility, multi-layer printed circuit boards with dedicated power and ground planes are typically used, which substantially increase the value of $C_{3}$ in the signal input path. As a result, $C_{3}$ may reach several times—or even exceed—the coupling capacitance $C_{1}$, and it is affected by factors such as the relative positions of the probe and measured cable, input capacitance of the operational amplifier in the circuit, PCB layout, and substrate material. Consequently, $C_{3}$ cannot be ignored and exerts a significant impact on the measurement results. Reference [19] proposed a self-calibration technique based on switch-controlled parameter correction, which eliminates the effect of $C_{3}$ by solving simultaneous equations. However, this approach overlooks the capacitance introduced by the switches themselves and the dynamic variations in coupling capacitance during switching, making it unsuitable for field engineering applications. Similarly, reference [13] experimentally demonstrated that, for high-precision measurements, calibrating and storing $V_{OR}$ when it acts solely on $C_{3}$ (denoted as $V_{zero}$) provides a direct and convenient method to compensate for its influence. Nevertheless, re-examining Equation (2) reveals that $V_{OR}$ results from the combined effects of $V_{R}$ acting on both $C_{3}$ and $C_{1}$. When $C_{3}$ is excessively large, small variations in $C_{1}$—caused, for instance, by minor cable displacement or temperature fluctuations—may become indistinguishable, leading to substantial measurement deviations.

For example, consider $C_{1}=3\;\mathrm{pf}$ (estimated using the capacitance definition formula) and $C_{3}=30\;\mathrm{pf}$ (an approximate value dependent on PCB layout an input capacitance of the operational amplifier). If $C_{1}$ varies slightly by 0.1pf (i.e., $\Delta C_{1}=0.1\;\mathrm{pf}$) due to cable movement, the resulting change in the measured voltage can be significant.

(10) $$\frac{\Delta V_{OX}}{V_{OX}}=\frac{\Delta C_{1}}{\Delta C_{1}+C_{1}}=\frac{0.1\;\mathrm{pf}}{3.1\;\mathrm{pf}}\approx 3.2\%$$

(11) $$\frac{\Delta V_{OR}}{V_{OR}}=\frac{\Delta C_{1}}{\Delta C_{1}+C_{1}+C_{3}}=\frac{0.1\;\mathrm{pf}}{33.1\;\mathrm{pf}}\approx 0.3\%$$

It is evident that as the distributed capacitance $C_{3}$ increases further, the value in Equation (11) decreases correspondingly. In the circuit, this phenomenon manifests as a pronounced change in $V_{OX}$, while the variation in $V_{OR}$ remains minimal or cannot be accurately detected, ultimately resulting in substantial measurement errors when using Equation (8) or (9).

(2) Phase drift of the mixed-frequency signal. In the signal injection scheme, the measured signal and the injected signal become aliased during transmission. Discretely sampling the signal and subsequently using digital filters combined with RMS calculations can greatly simplify the circuit and enable simultaneous measurement of electrical parameters such as frequency and phase. However, because the measured signal source and the injected signal source are independent, a low-frequency relative phase drift occurs during transmission and sampling [20]. When using the same reference clock for digital processing, sampling drift arises, causing fluctuations in the calculated results. Furthermore, the influence of capacitance in the transmission path may amplify the effect caused by phase drift, and this influence is difficult to eliminate using conventional digital filtering.

### 2.3. Proposed Solution

To address this problem, this paper proposes a comprehensive improvement scheme to address the aforementioned problems. First, an RC-type signal input circuit is designed. By employing an RC input, the influence of distributed capacitance is reduced, and the sensitivity of the injected signal to variations in coupling capacitance is enhanced. This ensures that even minor dynamic changes in coupling capacitance during long-term monitoring do not affect the measurement results. Second, the mechanism by which phase drift of the mixed-frequency signal causes data fluctuations is analyzed in depth. Combining the phase drift measurement method from [21] with the practical application scenario of this study, a method to increase the phase-shift vibration frequency is proposed. Through a non-integer harmonic injection strategy, the relative phase-shift vibration frequency of the mixed signal is artificially increased, enabling it to be effectively filtered out by subsequent digital filters. Consequently, while simplifying the analog circuit structure, the measurement accuracy is significantly improved. This work aims to provide a practical and feasible solution for high-precision, high-stability non-contact online voltage monitoring in complex electromagnetic environments.

## 3. Circuit Principles

In current engineering applications, online voltage monitoring devices predominantly employ micro controller units (MCU) as processing cores. Digital signal processing techniques are used to replace complex analog circuits and eliminate the need for computers in large-scale raw data processing. Measurement results are typically transmitted via a field bus interface. The verification circuit designed in this study adopts the same architecture to validate the proposed methodology.

Following the recommendation of Shenil et al. [12], the voltage sensing probe is implemented using a double-layer copper tube structure, as illustrated in Figure 1. The outer layer, with a diameter of 11 mm and a length of 40 mm, serves as the shielding layer, while the inner layer, with a diameter of 6 mm and a length of 4 mm, functions as the sensing electrode. The probe is connected to the conditioning circuit through a shielded cable to minimize interference and maintain signal integrity.

### Resistive-Capacitive Signal Input Circuit

**Figure 3:**
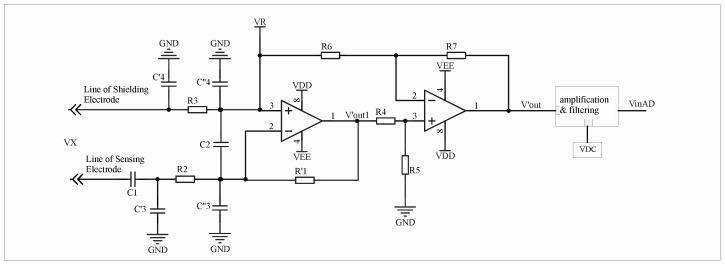
Equivalent Schematic Diagram of Resistive-Capacitive Input.

**Figure 4:**
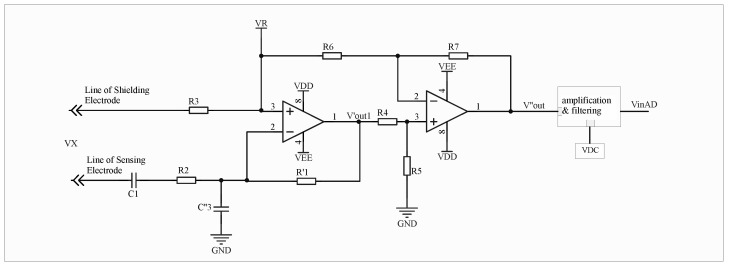
Simplified Equivalent Circuit Diagram of Resistive-Capacitive Input.

To minimize the influence of large distributed capacitance on the measurement results, this study introduces a resistive–capacitive signal input circuit. The proposed design effectively divides the distributed capacitance within the signal input loop into two distinct components, thereby reducing its impact on overall measurement accuracy. The schematic diagram of the circuit is shown in Figure 3:

The resistor is integrated into the signal input loop, and its resistance value must be carefully selected. If the resistance is too high, the effective signal entering the subsequent processing circuit will be excessively attenuated; conversely, if it is too low, the resistor will fail to perform its intended function. Based on existing studies and experimental validation, the influence of $C_{3}$ becomes nearly negligible after adopting the proposed resistive–capacitive input configuration. Consequently, the equivalent circuit shown in Figure 3 can be further simplified, shown in Figure 4:

At this point,

(12) $$V_{OX}^{\prime }=\frac{\omega_{x}R_{1}^{\prime }C_{1}}{1+\omega_{x}R_{2}C_{1}}V_{X}$$

(13) $$V_{OR}^{\prime }=\omega_{R}R_{1}^{\prime }\left(\frac{C_{1}}{1+\omega_{R}R_{2}C_{1}}+C_{3}^{\prime \prime }\right)$$

According to the circuit configuration, when $C_{1}=0$, the branch containing $C_{1}$ and $R_{2}$ can be regarded as an open circuit with respect to $V_{OR}^{\prime }$. Consequently, the measured value depends solely on $C_{3}^{\prime \prime }$, forming the Vzero term in Equation (5). Now

(14) $$V_{zero}^{\prime }=\omega_{R}R_{1}^{\prime }C_{3}^{\prime \prime }$$

(15) $$V_{OR2}^{\prime }=V_{OR}^{\prime }-V_{zero}^{\prime }=\frac{\omega_{R}R_{1}^{\prime }C_{1}}{1+\omega_{R}R_{2}C_{1}}$$

By appropriately selecting the parameters $R_{2}$ and $\omega_{R}$ such that $\omega_{R}R_{2}C_{1}\ll 1$, the algorithmic principles described in Section 3 remain valid for the improved resistive–capacitive input circuit. If $C3^{\prime \prime }$ remains constant, the measurement is independent of $C_{1}$ and is unaffected by its variations.

(16) $$V_{x}=\frac{V_{OX}^{\prime }\omega_{R}V_{R}}{V_{OR2}^{\prime }\omega_{x}}$$

Moreover, the introduction of $R_{2}$ effectively alleviates the original problem in which $V_{OR}$ exhibited weak sensitivity to variations in $C_{1}$ caused by a large $C_{3}$, since the effective input capacitance is now reduced to $C_{3}^{\prime \prime }$.

With the RC input configuration, the originally large input distributed capacitance is divided into two parts, thereby reducing the value of $V_{ZERO}$ to some extent. In the first prototype developed for this study, a multi-layer board structure was adopted to meet application requirements. When testing with the pure capacitive input structure described in literature [17], it was observed that while the input signal remained unchanged, slightly shifting the measured cable (i.e., altering the coupling capacitance) caused almost no variation in $V_{OR}$, whereas $V_{OX}$ changed significantly. This resulted in considerable measurement error. Based on this finding and subsequent theoretical analysis, this research proposes an improved RC input structure. The modified circuit resolves this issue, and the corresponding test data are provided in later sections. This demonstrates that the RC input configuration significantly reduces the system’s dependence on input distributed parameters, offering stable input conditions for long-term online voltage monitoring.

In practical circuits, component tolerances are inevitable. For example, the tolerance of the resistor *R* in the input loop can affect the time constant of the first-stage circuit to some degree. However, since the method presented here relies on ratio-based calculations and frequency-domain separation, resistor tolerances within a reasonable range do not significantly impact measurement accuracy. Discretization errors from batch-produced components can be addressed through calibration without requiring additional data processing. Furthermore, this study primarily focuses on low-voltage online monitoring. Should extension to higher voltages be necessary, a voltage-divider resistor can be added at the input stage following the approach outlined in literature [13].

## 4. Data Processing Methods

According to Equation (8), filters must be designed to separately extract $V_{OX}$ and $V_{OR}$ from $V_{OUT}$. As discussed earlier, the proposed method involves division operations with a relatively small denominator, thereby imposing stringent requirements on data stability. During data processing, in addition to conventional factors such as sampling rate, response time, and pass band error, particular attention must be given to phase drift in the mixed-frequency signal during transmission, as it can induce significant fluctuations in the measured data.

### 4.1. Causes of Data Fluctuations Due to Phase Drift in Mixed-Frequency Signals

In the non-contact voltage monitoring method proposed in this paper, a signal $V_{R}$ must be injected to enable real-time monitoring of the coupling capacitance. Consequently, the measured signal is superimposed with the injection signal and transmitted to the subsequent conditioning and processing circuits as a mixed-frequency signal for amplification, analog-to-digital (AD) sampling, and computation. To more intuitively illustrate the data fluctuation issue caused by phase drift in the mixed-frequency signal, let $S_{1}\left(t\right)$ and $S_{2}\left(t\right)$ denote the measured signal $V_{X}$ and the injected signal $V_{R}$, respectively.

(17) $$S_{1}\left(t\right)=A_{1}\cos\left(2\pi *f_{1}\left(t\right)+\varphi_{1}\left(t\right)\right)$$

(18) $$S_{2}\left(t\right)=A_{2}\cos\left(2\pi *f_{2}\left(t\right)+\varphi_{2}\left(t\right)\right)$$

In the equation, $f_{i}\left(t\right)$ represents the frequency of the measured signal and the injected signal, and $\varphi_{i}\left(t\right)$ represents the signal phase, where both signals may exhibit slight frequency drifts $\Delta f_{i}\left(t\right)$. The mixed-frequency signal resulting from the aliasing of $S_{1}\left(t\right)$ and $S_{2}\left(t\right)$ is processed through a linear conditioning circuit to obtain the signal x(t), whose relative phase drift

(19) $$\Delta \Phi \left(t\right)=2\pi *\left(f_{2}\left(t\right)-f_{1}\left(t\right)\right)+2\pi *\left(\Delta f_{2}\left(t\right)-\Delta f_{1}\left(t\right)\right)+C$$

(20) $$C=\left(\varphi_{10}+\theta_{1}\right)-\left(\varphi_{20}+\theta_{2}\right)$$

When the circuit structure is fixed, *C* can be considered a constant. However, in the circuit discussed in this paper, since the coupling capacitance may vary, *C* is not strictly constant. For the sake of simplified analysis, it is treated as a constant in this discussion. At this stage, x(t) is fed into the ADC for conversion into a discrete signal, followed by digital computation. Assuming the sampling period is $T_{S}$ and the number of sampling points per cycle is *n*, ΔΦ(t) can then be discretized as follows:

(21) $$\Delta \Phi \left(n\right)=2\pi *\left(f_{2}-f_{1}\right)nT_{S}+2\pi *\left(\Delta f_{2}-\Delta f_{1}\right)nT_{S}+C$$

For further analysis, by treating $S_{1}\left(t\right)$ as the reference signal, it follows that $\Delta f_{1}=0$ and $\Delta f_{2}-\Delta f_{1}=\delta f$. At this point,

(22) $$\Delta \Phi \left(n\right)=2\pi *\left(f_{2}-f_{1}\right)nT_{S}+2\pi *\delta fnT_{S}+C$$

It can be observed that, after the mixed-frequency signal is discretion, the presence of relative phase drift ΔΦ(n) introduces a sampling-sliding effect, which ultimately leads to fluctuations in the measurement results at the frequency corresponding to ΔΦ(n). If the injected signal is an integer harmonic of the measured signal, i.e., $f_{2}=kf_{1}$, then $2\pi \left(f_{2}-f_{1}\right)nT_{S}$ becomes a constant. Otherwise, it appears as a time-varying term whose fluctuation frequency depends on the deviation of $f_{2}$ from $kf_{1}$. Therefore, the fluctuation frequency of ΔΦ(n) can be analyzed using the following expression:

(23) $$f\left(\Delta \Phi \left(n\right)\right)=f_{2}-kf_{1}+\delta f$$

When $f_{2}=kf_{1}$, the frequency of ΔΦ(n) is determined by

(24) $$\Delta f_{2}-\Delta f_{1}=\delta f$$

**Figure 5:**
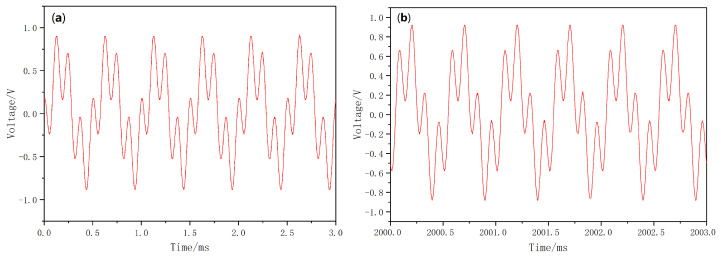
Waveform of Input Signal at AD Port under Integer Harmonic Injection: (**a**) t = 0–0.003 s, (**b**) t = 2–2.003 s.

This indicates that the relative phase ΔΦ(n) drifts at an extremely low frequency. A more detailed derivation of the formula is provided in Appendix A. Due to the sampling-sliding effect, the RMS measurement results fluctuate correspondingly at this same frequency. Moreover, when the value of *C* in Equation (22) approaches $\frac{\pi }{2}$ or $\frac{3\pi }{2}$, the amplitude of these fluctuations becomes more pronounced. Figure 5 illustrates the waveform of the mixed-frequency signal at the ADC input when $f_{2}=4f_{1}$. It can be observed that the waveforms in Figure 5a,b exhibit consistent phase alignment within each sub figure, yet a significant phase shift exists between them. In practice, experimental observations reveal that the phase drift is nearly imperceptible within a single oscilloscope sweep, but becomes clearly noticeable during long-term dynamic monitoring, thereby confirming the presence of phase drift with a relatively long drift period. The data in Figure 5 corresponds to the measured values of $V_{ADin}$, which represents the mixed signal of $V_{X}$ and $V_{R}$ at this stage. The waveform data was acquired using the oscilloscope’s storage function, with the oscilloscope set to a sampling rate of 500 KSa/s. A total of 1,048,554 data points (corresponding to 2.097 s) were recorded. Analysis of the entire dataset revealed noticeable drift in the data; however, within the captured period of slightly over 2 s, a complete cycle was not observed, indicating that the phase drift frequency is below 0.5 Hz. Due to the large volume of data, only a portion is presented here to illustrate the aforementioned theory. Figure 5a shows the waveform data from 0 to 0.003 s at the beginning, while Figure 5b displays the waveform data from 2 s to 2.003 s.

At this stage, the input mixed-frequency signal is subjected to discrete sampling and digital filtering, followed by RMS computation to obtain the measured voltage value. Figure 5 presents the measurement results when the target signal is 500 V at 2000 Hz, and the injected signal frequency is 8000 Hz. A total of 150 RMS samples were continuously acquired at a rate of one value per second. Apart from calibration, no additional data processing was applied following the internal computation.

**Figure 6:**
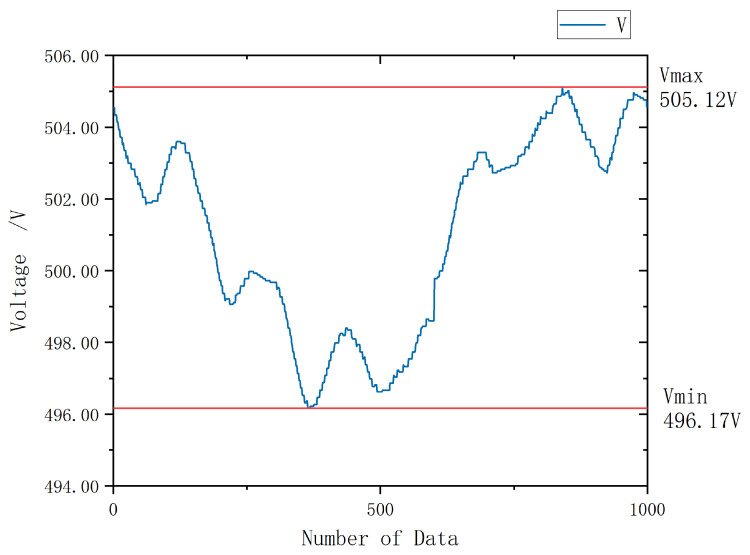
Schematic Diagram of Test Results under Integer Harmonic Injection.

As illustrated in Figure 6, the experimental data exhibit distinct fluctuations within an approximate range of [−1%, 1%], characterized by a relatively low fluctuation frequency. Although the amplitude of these variations appears small, they nonetheless represent a substantial deviation from the 0.2-class accuracy standard currently achievable by contact-type voltage monitoring systems. Furthermore, such low-frequency fluctuations are particularly difficult to suppress using conventional digital filtering techniques. This phenomenon cannot be attributed to spectral leakage or deficiencies in filter design. Control experiments conducted with $V_{X}=0$ and $V_{R}=0$ confirmed that individual signal inputs do not induce leakage into other frequency bands, and that RMS computations for single-frequency signals maintain excellent accuracy and linearity. However, when mixed-frequency signals are applied, the fluctuation phenomenon observed in Figure 6 emerges. Analysis of MCU memory data reveals that these fluctuations primarily stem from slight oscillations in $V_{OR}$ when $V_{X}\neq 0$. Although the amplitude of these oscillations is negligible relative to the RMS value of $V_{OR}$ itself, Equations (7) and (9) indicate that the final computation of $V_{X}$ depends solely on the residual component of $V_{OR}$ after subtracting $V_{zero}$—specifically, the value $V_{OR2}$ generated by $V_{R}$ acting on the coupling capacitance $C_{1}$. When $C_{3}\gg C_{1}$, the magnitude of $V_{OR2}$ becomes exceedingly small, rendering the calculation of $V_{X}$ highly sensitive to even minor fluctuations in $V_{OR}$, thereby resulting in pronounced oscillations in the computed results.

### 4.2. Method for Resolving Data Fluctuations Using Phase-Shift Vibration 

Based on the foregoing analysis, although the two frequency components of the mixed-frequency signal are inherently incoherent, they propagate through the same channel into the ADC stage and share a common sampling reference clock, rendering the relative phase drift ΔΦ(n) unavoidable. One potential solution is to employ analog filter circuits in combination with peak detection circuits to separately extract the amplitudes of the two signals before feeding them into the MCU for subsequent subtraction and division operations [16]. However, this approach makes it difficult to simultaneously obtain key parameters essential for online monitoring, such as frequency and phase. Moreover, it substantially increases circuit complexity, leading to higher implementation costs and reduced system reliability—factors that are particularly critical for large-scale engineering deployments. The method proposed in literature [21], which introduces a second frequency-band signal to measure phase drift in RF signals caused by system components, provides valuable inspiration. Based on this concept, and considering Equation (23) along with the frequency-adjustable characteristic of the injected signal employed in this study, a phase-shift vibration method is proposed. By intentionally offsetting the injected signal from the harmonic points of the measured signal by a certain frequency, the fluctuation of ΔΦ(n) is driven to a higher frequency. Combined with digital filtering algorithms, this approach effectively enhances the stability of data measurement results. The implementation is straightforward, significantly reducing the complexity of the overall scheme. For instance, when the measured signal frequency is $\omega_{x}=2000\;\mathrm{Hz}$ and the injection signal frequency is set to $\omega_{R}=8000\;\mathrm{Hz}$, Equation (27) indicates that the RMS value of $V_{R}$ will exhibit extremely low-frequency fluctuations (typically <1Hz). However, if the injection frequency is adjusted to $\omega_{R}=8500\;\mathrm{Hz}$, the fluctuation frequency of the $V_{R}$ RMS value increases significantly, as expressed by

(25) $$|f_{2}-kf_{1}+\delta f|=500\;\mathrm{Hz}$$

Fluctuations at this frequency can be readily attenuated using standard digital filtering techniques, thereby improving data stability and measurement precision.

**Figure 7:**
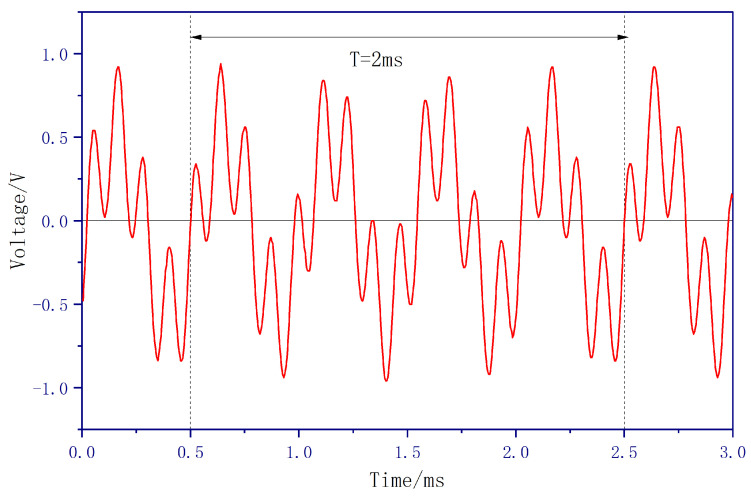
Waveform of Input Signal at ADC Port under Non-Integer Harmonic Injection.

The waveform of the mixed-frequency signal measured at the ADC input port under this non-integer harmonic injection condition is shown in Figure 7. As observed, the mixed-frequency signal exhibits a more pronounced phase drift, with a variation frequency of 500Hz, consistent with theoretical. The data in Figure 7 were acquired in the same manner as those in Figure 5. In this case, a phase drift period of 2 ms (frequency of 500 Hz) can be clearly observed.

**Figure 8:**
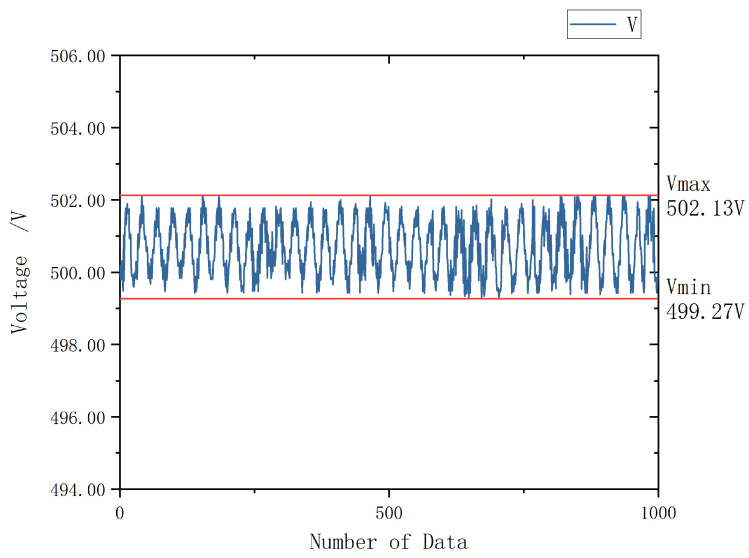
Schematic Diagram of Test Results under Non-Integer Harmonic Injection.

Applying the same data processing method used in Figure 6, the corresponding measurement results are presented in Figure 8.

From Figure 8, it can be observed that the stability of the improved data has been significantly enhanced, with the overall fluctuation range reduced to within 0.3%. This demonstrates the effectiveness of the proposed frequency deviation strategy in suppressing low-frequency oscillations and improving measurement consistency.

The phase-shift vibration method does not eliminate the relative phase drift itself. Instead, it alters its spectral distribution through frequency offset, shifting the originally low-frequency phase drift to a higher and controllable frequency band. Consequently, the slow quasi-static fluctuations in the RMS are transformed into periodic high-frequency vibrations. From a signal-processing perspective, such high-frequency vibrations can be effectively suppressed during RMS calculation using conventional low-pass filtering, without compromising the measurement response speed or increasing the complexity of the analog circuitry. Therefore, this method significantly improves numerical stability while maintaining the complete functionality of the measurement system.

## 5. Determination of Circuit Parameters

### 5.1. Selection of Injection Signal $V_{R}$

The frequency selection strategy for $V_{R}$ has been comprehensively discussed in the preceding chapter. According to [17], when $\omega_{R}\geq 10\;\mathrm{kHz}$, the capacitive reactance of the coupling capacitance between the sensing and shielding layers can no longer be neglected, and the virtual short assumption of the operational amplifier becomes invalid. As described in Section 3, although an excessively high frequency should be avoided, increasing the amplitude of $V_{R}$ is generally beneficial. One study [16] suggests that the optimal condition is achieved when $\omega_{R}V_{R}=\omega_{x}V_{x}$. However, as observed from the circuit schematic, the output of the first-stage operational amplifier, $V_{out1}^{\prime }$, contains a $V_{R}$ component. Therefore, when determining the amplitude of $V_{R}$, it is essential to ensure compatibility between $V_{out1}^{\prime }$ and the power supply voltage of the operational amplifier.

Furthermore, since $V_{R}$ is an internally injected signal, an excessively high amplitude may cause insulation degradation and induce electromagnetic interference on the measured signal through capacitive coupling. Considering these factors, this study conducted experiments on measured signals with frequencies of 50 Hz (500 V) and 2000 Hz (300 V). A DDS-based signal generation circuit—comprising an AD9833 digital signal generator and a digitally controlled potentiometer—was used to produce a sinusoidal AC signal $V_{R}$ with adjustable amplitude and frequency. For the 50 Hz test signal, injection frequencies of 2000 Hz and 1970 Hz were compared, while for the 2000 Hz signal, 8000 Hz and 8500 Hz were used. The amplitude of $V_{R}$ was adjusted such that the magnitudes of $V_{OX}$ and $V_{OR}$ in $V_{OUT^{\prime }}$ were approximately equal.

In the improved circuit proposed in this study, parameter determination must account for the input voltage range of the backend MCU to ensure distortion-free signal transmission throughout the entire signal chain. The STM32H7V7X6 micro controller (STMicroelectronics N.V., Geneva, Switzerland) was selected as the processing unit, implementing real-time monitoring for both 50 Hz and 2000 Hz signals using the following workflow: mixed-signal AC sampling → digital filtering → single-frequency RMS computation → algorithmic processing → bus communication output. The RMS values of the filtered signals were obtained using a “two-point multiplication plus low-pass filtering” method. The built-in general-purpose ADC of the STM32H7V7X6 accepts input signals within a 0–3.3 V range. To prevent the “floating ground” phenomenon caused by long signal loops, voltage measurements were taken at both the positive and negative terminals of the measured signal loop, with the true differential voltage obtained through a precision differential circuit. Circuit parameters were determined using a back-calculation approach, and the coupling capacitance $C_{1}$ was estimated based on the dielectric constant of commonly used cable materials. To ensure broad applicability and prevent data overflow during cable replacement, the coupling capacitance $C_{1}$ was set to 5 pf. The distributed capacitance $C_{3}^{\prime \prime }$ on the experimental prototype PCB was measured using an impedance bridge and found to be approximately 10 pf. When determining the key circuit parameters, the following design constraints must be satisfied:

1. According to standard engineering practice, a 20% safety margin should be reserved for the MCU input signal range. To simplify the hardware architecture, AC coupling is adopted for sampling. Consequently, the voltage at the ADC input must satisfy the following condition:
  (26) $$V_{ADinp}=V_{Xinp}+V_{Rinp}+V_{DC}\leq 80\%V_{ADmax}$$
  (27) $$V_{DC}=\frac{1}{2}V_{ADmax}$$
2. To simplify the circuit design and enhance reliability in engineering applications, the number of distinct power supply voltage levels for all electronic components should be minimized while still satisfying the operating requirements of standard industrial devices. In this design, the operational amplifiers along the signal path are powered by a 12 V supply, the MCU operates at 3.3 V, and the DC bias voltage $V_{DC}$ is set to 1.65 V.
3. At each stage of the operational amplifier circuits, the signal peak values must not exceed the corresponding supply voltage limits of the integrated amplifiers. In the improved circuit proposed in this paper, the stages most prone to exceeding these limits are the first-stage current-to-voltage (I–V) conversion circuit and the second-stage subtraction circuit, as both stages handle the full amplitude of $V_{R}$. Constrained by the frequency selection principles discussed in Section 4, the amplitude of $V_{R}$ should be as high as possible within the permissible range. As a result, the $V_{R}$ component appearing at the output of the first-stage I–V conversion circuit is substantially greater than the corresponding values of $V_{OR}$ and $V_{OX}$.

By substituting the relevant device parameters obtained from the three constraint conditions above into Equations (12) and (13), the prototype circuit parameters for this design can be determined. For the measured signal with a frequency of 50 Hz and an amplitude range of 0–500 V, the main circuit parameters are selected as Table 1:

The main circuit parameters selected for the 2000 Hz signal test are shown in Table 2:

### 5.2. Data Processing Method

This study primarily focuses on the accuracy and stability of online voltage monitoring. The data processing is centered on RMS calculation, which is performed using the relatively straight forward pointwise multiplication method. The data processing procedure is as follows: After applying the signal injection method, the signal input to the ADC is a mixed-frequency signal. Two band-pass filters are employed to separate the input signal sequence, allowing for the independent calculation of the RMS values of the measured signal $V_{OX}$ and the injected signal $V_{OR}$. The $V_{X}$ value is then obtained by calculation using Formulas (7) and (9). The $V_{zero}$ parameter in Formula (7) is acquired by setting $C_{1}$ to zero and is subsequently stored in a register for direct retrieval during computation. The extraction of $V_{OX}$ and $V_{OR}$ utilizes two band-pass filters, both implemented as 8th-order IIR filters. Their center frequencies and cutoff frequencies are determined by the measured signal and the injected signal. As indicated by the injection signal frequency selection method described earlier, a significant frequency gap is generally maintained between the injected signal and the measured signal. Therefore, the filter parameters can be designed using conventional methods. The principle of using the pointwise multiplication method to calculate the RMS value after the mixed signal is separated by the filters is as follows: When the RMS value exhibits fluctuations, the fluctuation term is denoted by a[n], where $a\left[n\right]=m\cos\left(2\pi f_{m}nT\right)$. Here, $f_{m}$ is the fluctuation frequency discussed in Section 4, and *m* is the modulation index (m≪1). The signal sampling sequence is given by:

(28) $$x\left[n\right]=A_{s}\left[1+a\left[n\right]\right]sin\left(2\pi fnT\right)$$

Pointwise multiplication of x[n] with itself yields:

(29) $$x^{2}\left[n\right]=A_{s}^{2}{\left(1+a\left[n\right]\right)}^{2}sin^{2}\left(2\pi fnT\right)$$

Expanding this gives:

(30) $$x{\left[n\right]}^{2}=\frac{A_{s}^{2}}{2}{\left(1+a\left[n\right]\right)}^{2}\left(1-cos\left(4\pi fnT\right)\right)$$

Since m≪1, we have ${\left(1+a\left[n\right]\right)}^{2}\approx 1+2a\left[n\right]$. Therefore:

(31) $$x^{2}\left[n\right]=\frac{A_{s}^{2}}{2}+A_{s}^{2}a\left[n\right]-\left(\frac{A_{s}^{2}}{2}+A_{s}^{2}a\left[n\right]\right)cos\left(4\pi fnT\right)$$

Clearly, $x^{2}\left[n\right]$ contains a DC component $\frac{A_{s}^{2}}{2}$, a componenty ($\frac{A_{s}^{2}}{2}+A_{s}^{2}a\left[n\right])cos\left(4\pi fnT\right)$ attwice the original signal frequency, and a fluctuation component ($A_{s}^{2}a\left[n\right]$) at frequency $f_{m}$. By passing $x^{2}\left[n\right]$ through a digital low-pass filter and then taking the square root of its output, the RMS value of x[n] can be obtained. Evidently, when $f_{m}$ is relatively high, it can be easily filtered out. However, if $f_{m}$ is extremely low, it becomes difficult to separate from the true RMS value. When employing the phase-shift vibration method, the cutoff frequency of the low-pass filter here should be set lower than the value of $f_{m}$, and the value of $f_{m}$ can be calculated using Equation (25).

## 6. Results

### 6.1. Test Conditions

The method proposed in this paper aims to address the challenges of non-contact online voltage monitoring. Two prototype devices were fabricated and evaluated using signal cables with cross-sectional areas of 4.0 mm^2^ and 2.0 mm^2^, respectively. The conducted experiments included full-scale accuracy tests, nonlinearity error assessments, and evaluations of the effects of displacement variations on measurement accuracy. Furthermore, both prototypes underwent a continuous 48-h powered operation test. During the experiments, the 4.0 mm^2^ cable was used for full-scale calibration. During testing, the ambient temperature was maintained at 25 ± 5 °C with a humidity of 75%, and the input distributed capacitance was kept constant throughout the test process. Different signal cables were replaced to simulate the variations in coupling capacitance that may occur from production to field application. Furthermore, due to the small aperture of the coupling probe, it was difficult to obtain precise measurements of cable displacement to determine the exact change in coupling capacitance. However, random displacement of small-diameter cables within the probe was used to simulate the dynamic variation of the coupling capacitance. The test voltage and cable information are shown in Table 3.

**Figure 9:**
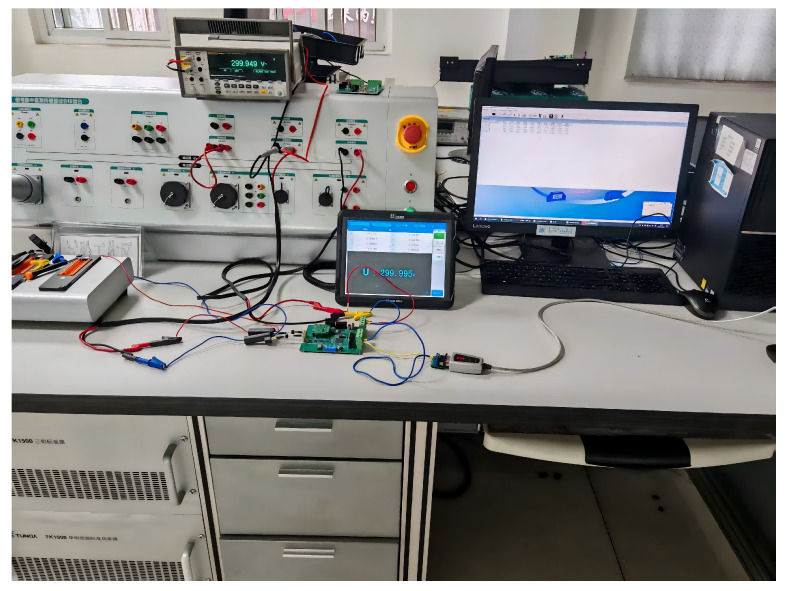
Experimental Setup Diagram.

During the experiments, a standard single-phase wide band AC voltage source with an accuracy class of 0.05 was used to generate the test signal. A 6.5-digit high-precision bench top multimeter was employed to monitor the output voltage, serving as the reference for comparison with the prototype measurement results, as shown in Figure 9.

### 6.2. Results Analysis

**Figure 10:**
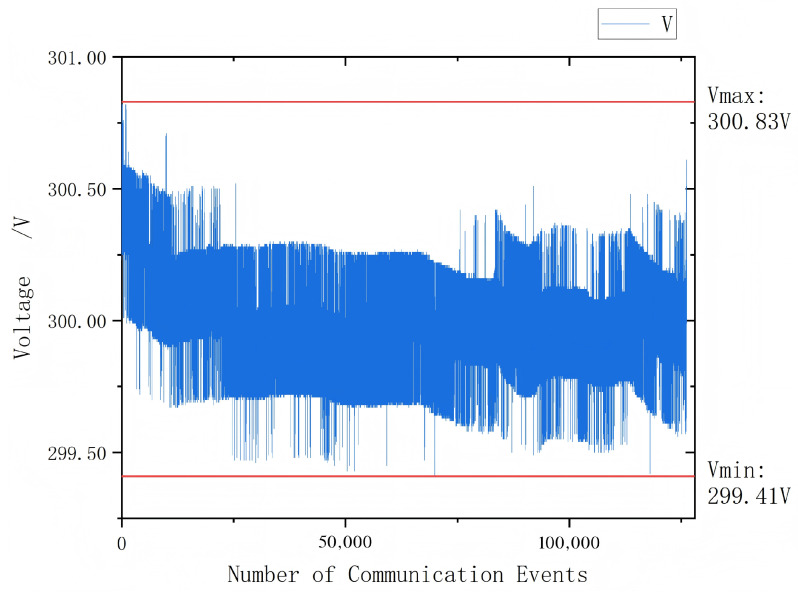
Long-term Communication Data.

The experimental results demonstrate that the proposed method achieves excellent linearity, with variations in cable cross-sectional area and conductor position exerting negligible influence on the measurement accuracy. Nonlinearity errors are shown in the Table 4 and Table 5:

The non-linearity error for 50 Hz, 50–500 V voltage is also within 0.3%. When repeated experiments were conducted at room temperature with small-diameter cables randomly positioned within the probe aperture, the standard deviation of 10 measurement results was less than 0.1 V under constant input signal conditions. This further verifies that in the signal injection-based non-contact voltage measurement method, the measurement results are virtually unaffected by coupling capacitance. It should be noted that all prototypes in this experiment were calibrated using their own data; therefore, component discretization errors do not impact the assessment of sensor accuracy and linearity. To further evaluate its suitability for online monitoring applications, a continuous long-term power-on test was performed. Data acquisition was implemented via RS485 bus communication, with the host computer configured to sample data at 1-s intervals and a baud rate of 19,200 bps. The host computer functioned solely as a data logger and display terminal, without any additional data processing. The results of the long-term stability test are presented in Figure 10.

**Figure 11:**
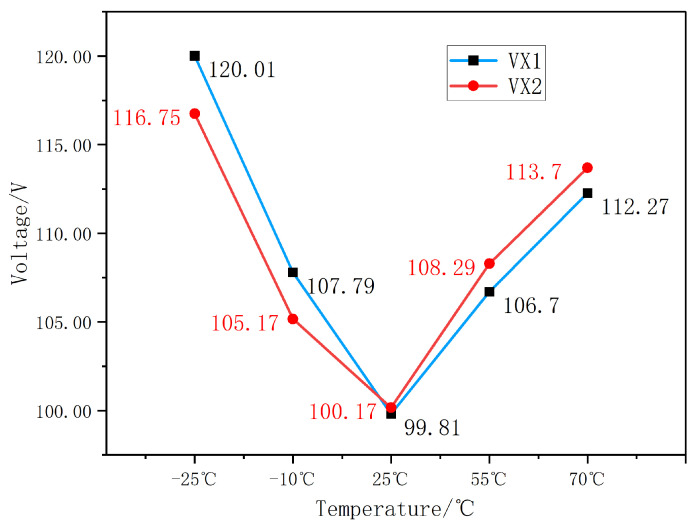
Variation of Output Voltage with Temperature.

The test results show slight initial drift upon power-on, after which the measurements remain highly stable during prolonged operation. The initial drift may be attributed to operational heating, suggesting that the method is relatively sensitive to temperature. To investigate this issue, two prototypes were tested at −25 ℃, −10 °C, 25 °C, 55 ℃ and 70 °C, respectively. During testing, each prototype was placed in a temperature chamber and allowed to stabilize for 2 h at each temperature point before readings were taken. To facilitate comparison, both prototypes were supplied with an input signal of 100 V at 2000 Hz. The measurement data are shown in Figure 11.

As can be seen from Figure 11, the output Voltage of the two prototypes follow similar trends with temperature variation, but the rate of change with temperature is not identical for each unit. Therefore, to achieve better temperature performance, individual piecewise compensation would be required for each prototype. In this study, a piecewise linear compensation method was applied to both prototypes individually, successfully reducing the temperature drift to within 3%. However, the compensation coefficients differed between the two prototypes, which may increase time costs during mass production. In fact, the research team has taken note of this phenomenon and has initiated a systematic study on the factors influencing the temperature characteristics of the sensor and on improving its temperature adaptability. This work is currently in progress.

## 7. Discussion

This paper presents an improved non-contact online voltage monitoring method designed to meet practical engineering requirements. A resistive–capacitive input structure is introduced, compared with existing studies, and this work addresses the issue of increased measurement error in traditional electric-field-coupling-based non-contact voltage measurement methods using signal injection, where an excessively large input distributed capacitance leads to insensitivity of the injected signal to subtle changes in coupling capacitance. It thereby relaxes the requirements for PCB layout and expands the applicable scope. Meanwhile, a phase-shift vibration method is proposed, which employs non-integer harmonic injection to resolve the problem of low-frequency data fluctuations caused by relative phase drift of the mixed-frequency signal. This further mitigates potential data-stability issues in existing research. Effectiveness of the proposed approach in improving measurement accuracy is verified through tests on low-frequency (<2000 Hz) and low-voltage (<500 V) signals. Experimental results show that long-term online monitoring at room temperature achieves class 0.5 accuracy, and the influence on measurement results due to cable replacement or minor displacement of the conductor through the probe is minimal, meeting the requirements for remote online monitoring of low-frequency, low-voltage signals under room-temperature conditions. Nevertheless, it was also observed during the experiments that the method requires temperature compensation for applications that must operate across a wide temperature range, such as outdoor monitoring. Conducting an in-depth analysis of the factors affecting the temperature characteristics of this method to achieve better thermal performance will constitute the focus of the next research phase.

## Figures and Tables

**Table 1:** Parameter table when the measured signal is 50 Hz.

$V_{R}$ (V)	$\omega_{R}$ (Hz)	$R_{1}^{\prime }\;\left(\Omega \right)$	$R_{2}\;\left(\Omega \right)$	Gain ^1^
7	2000	100k ± 1%	1M ± 1%	10
7	1970	100k ± 1%	1M ± 1%	10

^1^ Gain is the gain of the conditioning circuit between $V_{OUT}$ and $V_{AD}$.

**Table 2:** Parameter table when the measured signal is 2000 Hz.

$V_{R}$ (V)	$\omega_{R}$ (Hz)	$R_{1}^{\prime }\;\left(\Omega \right)$	$R_{2}\;\left(\Omega \right)$	Gain ^1^
4	8000	40k ± 1%	1M ± 1%	10
4	8500	40k ± 1%	1M ± 1%	10

^1^ Gain is the gain of the conditioning circuit between $V_{OUT}$ and $V_{AD}$.

**Table 3:** Benchmark Test Conditions.

$V_{X}$ (V)	$\omega_{X}$ (Hz)	Cross-Sectional Area of the Measured Signal Cable
0–500	50	4.0 mm^2^
0–500	50	2.0 mm^2^
0–300	2000	4.0 mm^2^
0–300	2000	2.0 mm^2^

**Table 4:** Measurement Data of Voltage Linearity at 2000 Hz, 30–300 V.

Theoretical Value (V)	Measured Value (V)	Error (%)
30	30.05	0.02
60	60.15	0.05
90	89.99	0.00
120	120.39	0.13
150	150.14	0.05
180	180.42	0.14
210	209.86	0.06
240	240.33	0.11
270	270.35	0.13
300	300.14	0.05

**Table 5:** Measurement Data of Voltage Linearity at 50 Hz, 50–500 V.

Theoretical Value (V)	Measured Value (V)	Error (%)
50	49.88	−0.25
100	100.24	0.24
150	149.81	−0.13
200	199.70	−0.15
250	250.24	0.10
300	300.54	0.18
350	350.50	0.14
400	400.59	0.15
450	450.74	0.16
500	500.49	0.10

## Data Availability

The original contributions presented in this study are included in the article. Further inquiries can be directed to the corresponding authors.

## Abbreviations

The following abbreviations are used in this manuscript:

PCB	Printed Circuit Board
MCU	Micro-controller Unit
RMS	Root Mean Square

## Appendix A. Causes of Data Fluctuations Due to Phase Drift in Mixed-Frequency Signals

Detailed Derivation Process

(A1) $$S_{1}\left(t\right)=A_{1}\cos\left(2\pi *f_{1}\left(t\right)+\varphi_{1}\left(t\right)\right)$$

(A2) $$S_{2}\left(t\right)=A_{2}\cos\left(2\pi *f_{2}\left(t\right)+\varphi_{2}\left(t\right)\right)$$

where $f_{1}\left(t\right)$ and $f_{2}\left(t\right)$ denote the frequencies of the measured and injected signals, respectively, both of which may exhibit slight deviations. $\varphi_{i}\left(t\right)$ represents the phase of each signal. In practical systems, since $S_{1}\left(t\right)$ and $S_{2}\left(t\right)$ originate from independent signal sources, their phase drift, $\varphi_{1}\left(t\right)$ and $\varphi_{2}\left(t\right)$, are uncorrelated. As a result, the mixed-frequency signal S(t) exhibits the characteristics of a non-stationary signal.

(A3) $$\varphi_{i}\left(t\right)=\varphi_{i0}+2\pi *\Delta f_{i}\left(t\right)$$

where $\varphi_{i0}$ denotes the initial phase of the signal, and $\Delta f_{i}\left(t\right)$ represents the deviation of the signal frequency from its ideal value (typically less than 1 Hz when using high-precision signal sources).

As illustrated in Figure 4, the signal after the first-stage operational amplifier is a mixed-frequency signal generated by the aliasing of $S_{1}\left(t\right)$ and $S_{2}\left(t\right)$. This signal is subsequently passed through a series of linear conditioning circuits for amplification, filtering, and other processing, ultimately producing x(t), which is then subjected to analog-to-digital conversion and computational analysis.

(A4) $$x\left(t\right)=A_{1}^{\prime }\cos\left(2\pi *f_{1}\left(t\right)+\Phi_{1}\left(t\right)\right)+A_{2}^{\prime }\cos\left(2\pi *f_{2}\left(t\right)+\Phi_{2}\left(t\right)\right)$$

(A5) $$\Phi_{1}\left(t\right)=\varphi_{1}\left(t\right)+\theta_{1}=\varphi_{10}+2\pi *\Delta f_{1}\left(t\right)+\theta_{1}$$

(A6) $$\Phi_{2}\left(t\right)=\varphi_{2}\left(t\right)+\theta_{2}=\varphi_{20}+2\pi *\Delta f_{2}\left(t\right)+\theta_{2}$$

where $A_{1}^{\prime }$ and $A_{2}^{\prime }$ denote the amplitudes of the two frequency components in the mixed-frequency signal after passing through the analog channel, while $\theta_{1}$ and $\theta_{2}$ represent the phase delays introduced by the channel capacitance, which can be regarded as constant for simplified analysis. Since the system is linear, the phases of the two signals at different frequencies remain relatively independent, and their relative phase drift can be expressed as:

(A7) $$\Delta \Phi \left(t\right)=\left(2\pi *f_{1}\left(t\right)+\varphi_{10}+2\pi *\Delta f_{1}\left(t\right)+\theta_{1}\right)-2\pi *f_{2}\left(t\right)+\varphi_{20}+2\pi *\Delta f_{2}\left(t\right)+\theta_{2})$$

Simplification yields:

(A8) $$\Delta \Phi \left(t\right)=2\pi *\left(f_{2}\left(t\right)-f_{1}\left(t\right)\right)+2\pi *\left(\Delta f_{2}\left(t\right)-\Delta f_{1}\left(t\right)\right)+C$$

(A9) $$C=\left(\varphi_{10}+\theta_{1}\right)-\left(\varphi_{20}+\theta_{2}\right)$$

When the circuit structure is fixed, *C* can be considered a constant. However, in the circuit discussed in this paper, since the coupling capacitance may vary, *C* is not strictly constant. For the sake of simplified analysis, it is treated as a constant in this discussion. At this stage, x(t) is fed into the ADC for conversion into a discrete signal, followed by digital computation. Assuming the sampling period is $T_{S}$ and the number of sampling points per cycle is *n*, ΔΦ(t) can then be discretized as follows:

(A10) $$\Delta \Phi \left(n\right)=2\pi *\left(f_{2}-f_{1}\right)nT_{S}+2\pi *\left(\Delta f_{2}-\Delta f_{1}\right)nT_{S}+C$$

For further analysis, by treating $S_{1}\left(t\right)$ as the reference signal, it follows that $\Delta f_{1}=0$ and $\Delta f_{2}-\Delta f_{1}=\delta f$. When the frequency settings of the measured and injected signals remain constant, δf is an extremely small quantity (typically less than 1 Hz when using high-precision signal sources) and is not fixed, as it primarily depends on the relative frequency drift between the two signal sources. At this point,

(A11) $$\Delta \Phi \left(n\right)=2\pi *\left(f_{2}-f_{1}\right)nT_{S}+2\pi *\delta fnT_{S}+C$$

It can be observed that, after the mixed-frequency signal is discretion, the presence of relative phase drift ΔΦ(n) introduces a sampling-sliding effect, which ultimately leads to fluctuations in the measurement results at the frequency corresponding to ΔΦ(n). According to Equation (24), if the injected signal is an integer harmonic of the measured signal, i.e., $f_{2}=kf_{1}$, then $2\pi \left(f_{2}-f_{1}\right)nT_{S}$ becomes a constant. Otherwise, it appears as a time-varying term whose fluctuation frequency depends on the deviation of $f_{2}$ from $kf_{1}$. Therefore, the fluctuation frequency of ΔΦ(n) can be analyzed using the following expression:

(A12) $$f\left(\Delta \Phi \left(n\right)\right)=f_{2}-kf_{1}+\delta f$$

When $f_{2}=kf_{1}$, the frequency of ΔΦ(n) is determined by

(A13) $$\Delta f_{2}-\Delta f_{1}=\delta f$$

This indicates that the relative phase ΔΦ(n) drifts at an extremely low frequency. Due to the sampling-sliding effect, the RMS measurement results fluctuate correspondingly at this same frequency. Moreover, when the value of *C* in Equation (21) approaches $\frac{\pi }{2}$ or $\frac{3\pi }{2}$, the amplitude of these fluctuations becomes more pronounced.
